# What Is in a Name?

**Published:** 1897-11

**Authors:** 


### What is in a Name?

Sometimes this is difficult to determine. We are impelled to speak of this because written names come to us that are difficult and sometimes impossible to decipher, if dependence is had upon the chirography alone.

It is difficult to conceive why some persons will persist in writing words and especially the writer's name, in such a manner as to be illegible, or even though not wholly so, but of such character as to require time for the discovery of the name.

Now, to all persons thus afflicted, we would suggest either learn to write legibly, or use a type-writer, or secure a legible writer to do your work, even to writing your name. The owner of the name can always make it valid, though some one else wrote it, by attaching his mark to it. We object to all illegible writing, but especially to illegible names.

This is a very desirable feature of manual education: to write a clear and readable hand.

